# Comparative Study on Statistical-Variation Tolerance Between Complementary Crossbar and Twin Crossbar of Binary Nano-scale Memristors for Pattern Recognition

**DOI:** 10.1186/s11671-015-1106-x

**Published:** 2015-10-16

**Authors:** Son Ngoc Truong, SangHak Shin, Sang-Don Byeon, JaeSang Song, Hyun-Sun Mo, Kyeong-Sik Min

**Affiliations:** School of Electrical Engineering, Kookmin University, 77, Jeongneung-ro, Seongbuk-gu, Seoul 136-702 South Korea

**Keywords:** Statistical-variation tolerance, Complementary crossbar, Twin crossbar, Binary memristors, Memristor array, Pattern recognition

## Abstract

This paper performs a comparative study on the statistical-variation tolerance between two crossbar architectures which are the complementary and twin architectures. In this comparative study, 10 greyscale images and 26 black-and-white alphabet characters are tested using the circuit simulator to compare the recognition rate with varying statistical variation and correlation parameters.

As with the simulation results of 10 greyscale image recognitions, the twin crossbar shows better recognition rate by 4 % on average than the complementary one, when the inter-array correlation = 1 and intra-array correlation = 0. When the inter-array correlation = 1 and intra-array correlation = 1, the twin architecture can recognize better by 5.6 % on average than the complementary one.

Similarly, when the inter-array correlation = 1 and intra-array correlation = 0, the twin architecture can recognize 26 alphabet characters better by 4.5 % on average than the complementary one. When the inter-array correlation = 1 and intra-array correlation = 1, the twin architecture is better by 6 % on average than the complementary one. By summary, we can conclude that the twin crossbar is more robust than the complementary one under the same amounts of statistical variation and correlation.

## Background

Memristors are resistive memories which are based on either interface-switching [1, 2] or filamentary-switching mechanism [3–8]. In the interface-switching mechanism, the interface between the low-resistance region and high-resistance region can be moved according to an applied voltage or current [1, 2]. By doing so, we can control memristance gradually to store analogue value on memristors [1, 2]. However, the materials that show the interface-switching behaviours are rare, and the accuracy in controlling the memristance value is still considered a big concern to be used in practical applications [9]. Moreover, even a small amount of memristance variation can degrade the overall accuracy severely in analogue memristor-based neuromorphic systems [9].

Generally, most of the memristors are known to usually demonstrate the filamentary-switching behaviours, not the interface switching [3–8]. The filamentary-switching memristors can have either a high-resistance state (HRS) or a low-resistance state (LRS) [3–8]. With two states, we can store ‘1’ and ‘0’ on filamentary-switching binary memristors [3–8]. In addition to the fact that filamentary-switching materials are popular, binary memristors with the filamentary switching can be much more tolerant against statistical variations compared to analogue memristors with the interface switching [9]. This is due to the fact that HRS can still be much higher than LRS, in spite of large amounts of statistical variation in LRS and HRS [9]. Thus, for simple neuromorphic applications such as pattern recognition, binary memristor crossbar can be more useful and robust than analogue memristor crossbar in terms of material availability, statistical-variation tolerance, etc. [9].

For implementing the crossbar circuit of pattern recognition, the input image should be compared with the stored images which are already stored in the crossbar. By doing so, the crossbar circuit can calculate amounts of similarity between one input image and many stored images one by one [9, 10]. After comparing the amounts of similarity in the crossbar, the winner-take-all circuit chooses which one the best matches with the input image among many stored images in the crossbar [9, 10].

**Fig. 1:**
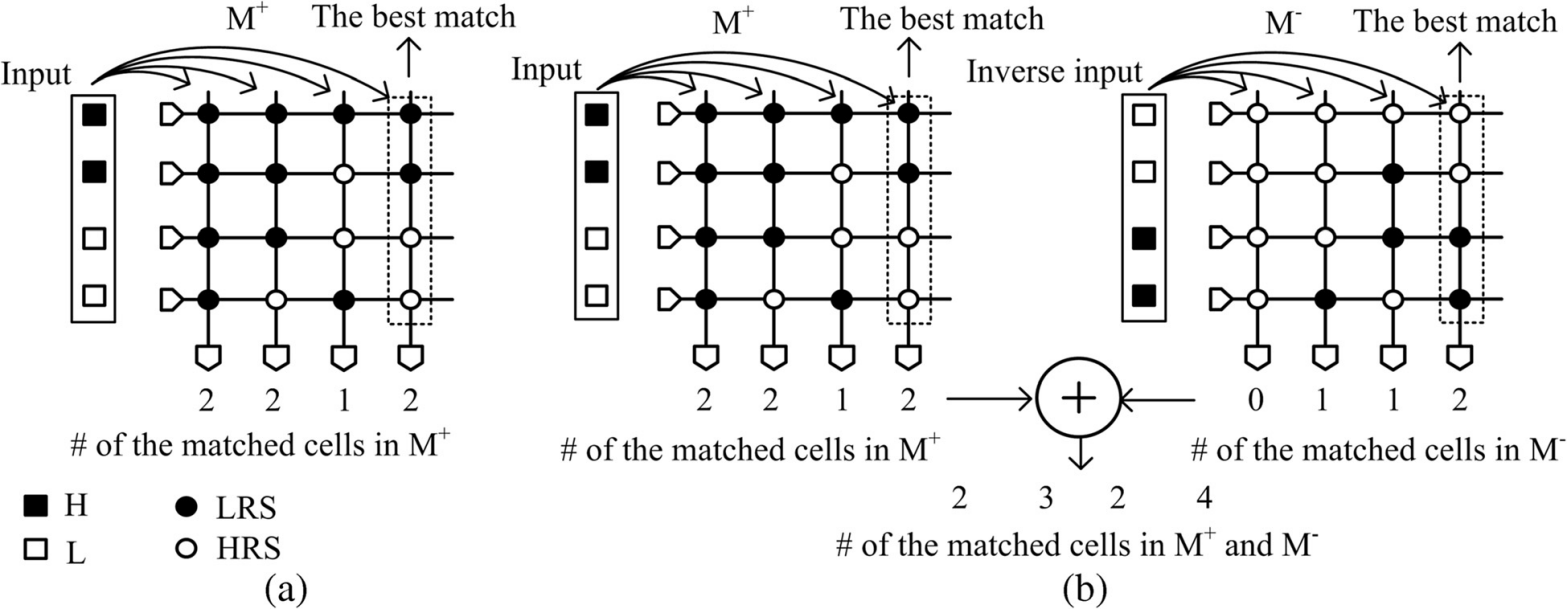
The operation of crossbar circuits of binary memristors for pattern recognition with **a** one crossbar array and **b** two crossbar arrays

The detailed operation of crossbar circuit which can perform pattern recognition is explained in Fig. 1a. Here, the solid box represents the input ‘H’ voltage and the open box represents the input ‘L’. Similarly, the solid circle in the array represents the stored ‘LRS’ data and the open circle represents the stored ‘HRS’. If the input ‘H’ is applied to the ‘LRS’ cell, it means that the input pixel matches with the stored data. In Fig. 1a, the input vector of ‘HHLL’ is compared with the four columns which are ‘LRS-LRS-LRS-LRS’, ‘LRS-LRS-LRS-HRS’, ‘LRS-HRS-HRS-LRS’ and ‘LRS-LRS-HRS-HRS’. As you see in Fig. 1a, the fourth column exactly matches with the input vector. However, here, the first, second and fourth columns show the same number of matched cells, as shown in Fig. 1a. The number of matched cells for each column is shown below the array of M^+^ in Fig. 1a. Thus, we cannot decide which column is the best match with the input vector in Fig. 1a.

To solve this problem, we have to use two crossbar arrays, not one, as shown in Fig. 1b [9]. Here, the input vector is applied to M^+^ and its inversion is applied to M^−^ at the same time [9]. The number of the matched cells to the input vector and its inversion is calculated by adding two numbers of the matched cells in M^+^ and M^−^ arrays. Hence, we can decide the best matched column with the input vector among four columns. In Fig. 1b, the best matched column with the input vector of ‘HHLL’ is the fourth column with ‘LRS-LRS-HRS-HRS’. The number of matched cells is as large as 4 for the fourth column.

To design the crossbar circuit with two binary memristor arrays, we can consider two types of crossbar architecture. The first architecture is the complementary crossbar in Fig. 1b [11, 12], and the other one is called the twin crossbar [10]. In this paper, we perform a comparative study between the two crossbar architectures for different variation and correlation parameters using the Monte Carlo method. Based on the results of this comparative study, we can estimate which crossbar architecture is better and how much it is better in various conditions of statistical variation and correlation.

## Methods

### Two Crossbar Architectures Simulated: Complementary Versus Twin Architectures

**Fig. 2:**
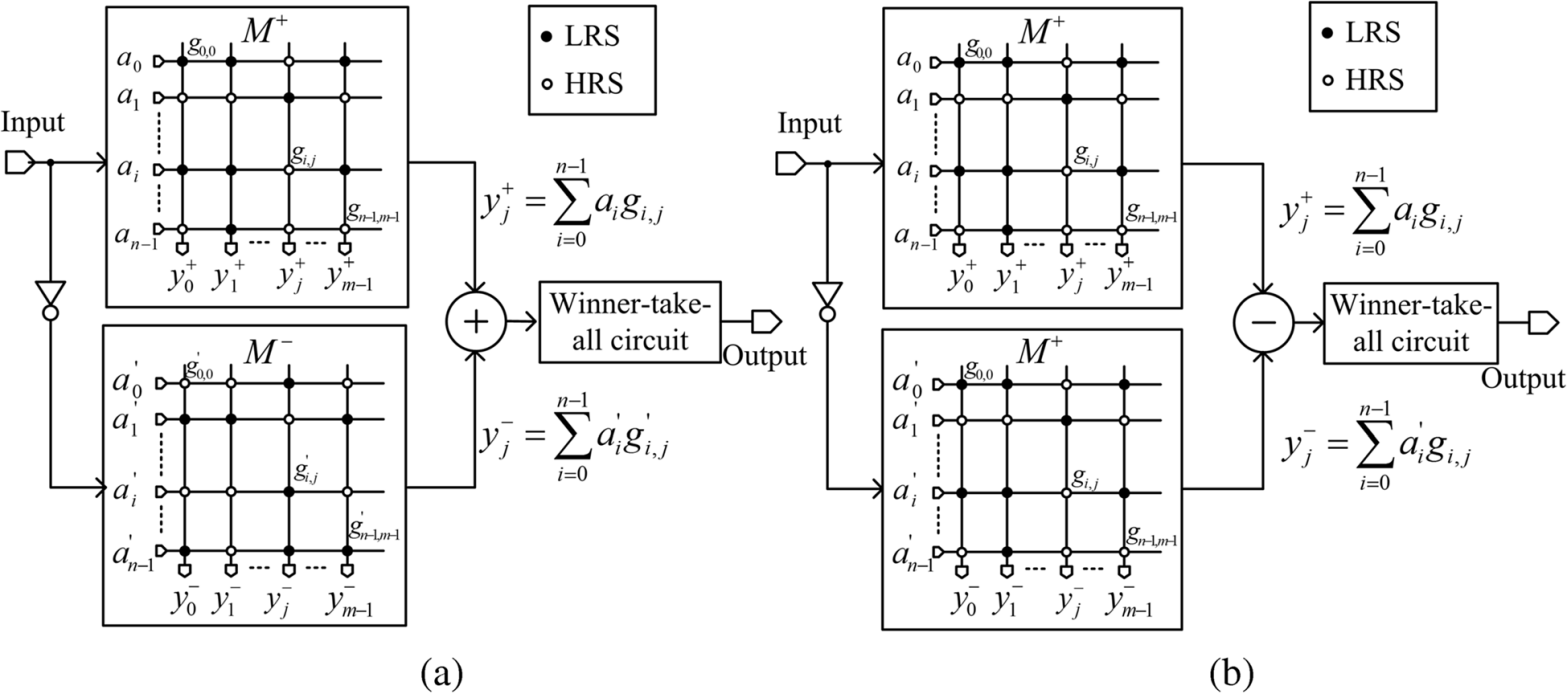
The crossbar array architectures of binary memristors for pattern recognition. **a** The complementary crossbar architecture [11, 12]. **b** The twin crossbar architecture [10]

Figure 2a shows the complementary crossbar architecture which is composed of two memristor arrays of M^+^ and M^−^ [11, 12]. The M^−^ array in Fig. 2a is the inversion of M^+^ array. Here, *a*_0_ is the input to the first row in M^+^ array. *a*_*n*−1_ is the input to the (*n*−1)th row in M^+^ array. *g*_0,0_ is the cross-point memristor conductance between the first row and first column in M^+^ array. *g*_*n*−1,*m*−1_ is the cross-point conductance between the (*n*−1)th row and (*m*−1)th column in M^+^ array. In binary memristors, memristor conductance can be either LRS or HRS. In Fig. 2a, LRS is represented by solid circles and HRS is represented by open circles. *a*^’^_0_ that is the inversion of *a*_0_ is applied to M^−^ array. Similarly, *g*^’^_0,0_ is the inversion of *g*_0,0_ in M^−^ array. *y*^+^_0_ is the output of the first column in M^+^ array and y^−^_0_ is the output of M^−^ array. *y*_0_ (*y*^+^_0_ and *y*^−^_0_) is the amount of similarity between the input vector and the first column in the crossbar. Similarly, *y*_*j*_ is the result of matching of the input vector with the *j*th column. *y*_*j*_ can be calculated as follows [10]:

1 $$ {y}_j={y}_j^{+}+{y}_j^{-}={\displaystyle \sum_{i=0}^{n-1}\left({a}_i{g}_{i,j}+{a}_i^{\hbox{'}}{g}_{i,j}^{\hbox{'}}\right)}. $$

Equation 1 is based on the exclusive NOR function that can calculate the amount of similarity between the input vector and the stored data [10]. If the input from *a*_0_ to *a*_*n*−1_ is very similar with the stored column from *g*_0,*j*_ to *g*_*n*−1,*j*_, *y*_*j*_ becomes large. On the contrary, if the input vector is very different from the stored column vector, *y*_*j*_ becomes small. All *y*_*j*_ values from *y*_0_ to *y*_*m*−1_ are compared with each other in the winner-take-all circuit [9, 10]. The largest *y*_*j*_ among all the *y*_*j*_ values from *y*_0_ to *y*_*m*−1_ is chosen by the winner-take-all circuit [9, 10].

To implement the crossbar circuit of pattern recognition, we can consider the other architecture different from the complementary one in Fig. 2a. Figure 2b shows the twin crossbar architecture with two identical M^+^ arrays of binary memristors [10]. Here, the upper M^+^ array is identical with the lower M^+^ array. The twin crossbar architecture was previously proposed by S. N. Truong et al., for low-power image recognition, using the discrete cosine transformation [10]. The twin crossbar architecture is based on Eq. 2 which also performs the exclusive NOR function like Eq. 1 [10].

2 $$ {y}_j={y}_j^{+}-{y}_j^{-}={\displaystyle \sum_{i=0}^{n-1}\left({a}_i{g}_{i,j}-{a}_i^{\hbox{'}}{g}_{i,j}\right)}. $$

One distinctive point of Fig. 2b from Fig. 2a is that the twin crossbar does not need to use the complementary M^+^ and M^−^ arrays [9, 10]. Instead of using M^+^ and M^−^ arrays in Fig. 2a, the twin crossbar can use only two identical M^+^ arrays, as shown in Fig. 2b [10]. By doing so, in the twin crossbar architecture, the number of LRS cells can be minimized using some image-processing algorithms such as DCT, because two arrays are identical [10]. One more thing to note here is that the addition of *y*^+^_*j*_ and *y*^−^_*j*_ in Fig. 2a is replaced with the subtraction of *y*^+^_*j*_ and *y*^−^_*j*_ in Fig. 2b [10]. Here, the subtraction in Fig. 2b can be easily implemented using the current mirror circuits [10].

These two different points between the complementary crossbar and the twin one can affect the statistical-variation tolerance of the binary memristor array. To analyse the tolerance quantitatively, this paper performs a comparative study between the two crossbar architectures which are shown in Fig. 2a, b, respectively, for different variation and correlation parameters using the Monte Carlo method. Based on the results of this comparative study, we can estimate which crossbar architecture is better and how much it is better in various conditions of statistical variation and correlation.

### Simulation Set-Up

In this paper, the memristor CMOS hybrid circuits were simulated by Cadence Spectre [13]. Here, memristors were modelled by Verilog-A [14, 15], and CMOS SPICE parameters were obtained from Samsung 0.13-μm CMOS technology. Here, HRS and LRS are assumed 100 MΩ and 10 kΩ, respectively. The supply voltage (*V*_DD_) is 1.0 V. The parameters that are used in the statistical simulation with the Monte Carlo method are listed in Table 1.

**Table 1:** The parameters that are used in the statistical simulation in this paper

Parameters used in the statistical simulation	Complementary crossbar [11, 12]	Twin crossbar [10]
HRS	100 MΩ	100 MΩ
LRS	10 kΩ	10 kΩ
Input voltage (*V*)	1	1
Number of iterations in the Monte Carlo simulation	1000	1000
Percentage variation in memristance	10–40 %	10–40 %
Inter-array correlation	0 or 1	0 or 1
Intra-array correlation	0 or 1	0 or 1

**Fig. 3:**
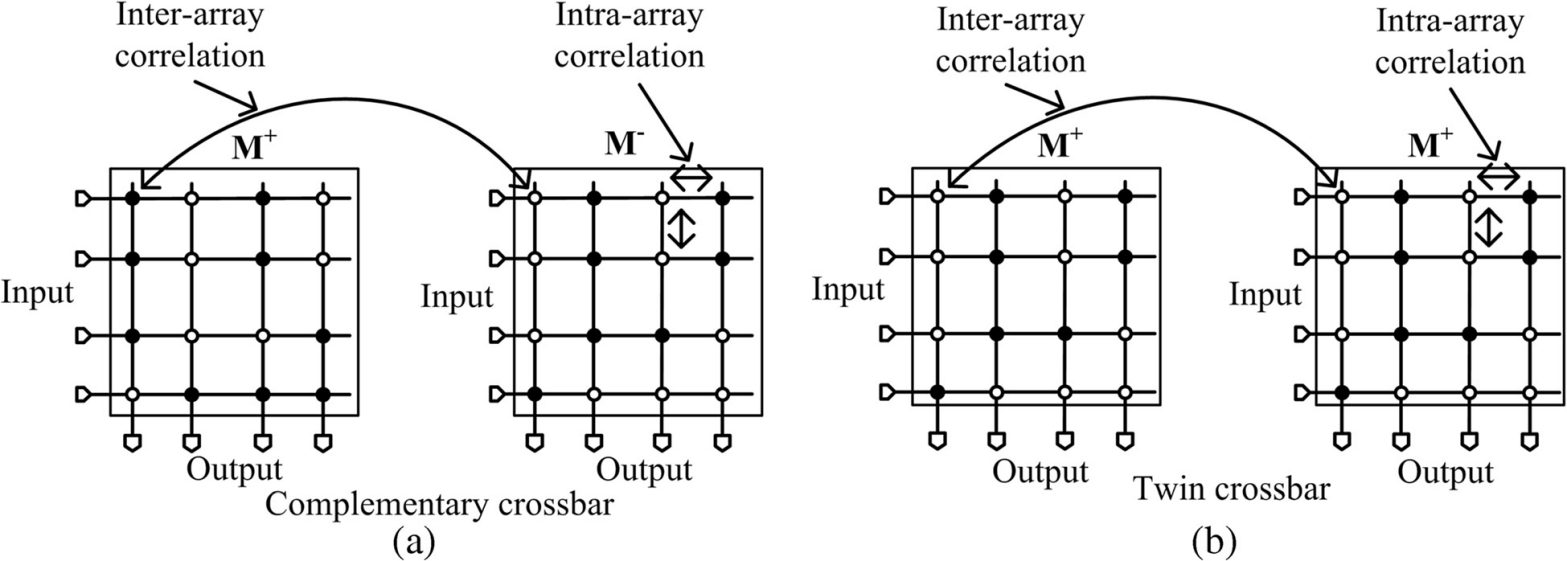
Inter-array correlation and intra-array correlation in **a** the complementary crossbar architecture and **b** the twin crossbar architecture

The statistical simulation was performed using the Monte Carlo method by Cadence Spectre [13]. Here, the percentage variation in memristance was assumed from 10 to 40 %, as shown in Table 1. The statistical distribution function is assumed the Gaussian distribution function. Another important parameter in the statistical simulation is the correlation. As indicated in Fig. 2a, the complementary crossbar has two M^+^ and M^−^ arrays, where M^−^ is the inversion of M^+^ array. With two M^+^ and M^−^ arrays, we can think of both intra-array correlation and inter-array correlation in performing the statistical simulation. Figure 3a shows the inter-array correlation and intra-array correlation in the complementary crossbar, where M^+^ array and M^−^ array are complementary with each other. If the correlation value is 1, it means that all the elements are varied in the same way. When the correlation is 0, all the elements are varied in random. Figure 3b shows the inter-array and intra-array correlations for the twin crossbar which is composed of two identical M^+^ arrays instead of using M^+^ and M^−^ arrays.

Actually, the inter-array and intra-array correlations that are mentioned here are originated from the die-to-die (inter-die) and within-die (intra-die) parameter fluctuations that are very well-known statistical variations that can be found in the semiconductor manufacturing process [16]. The inter-die parameter fluctuations which are caused by various variations of lot-to-lot, wafer-to-wafer and die-to-die can affect every element on a chip equally [16]. In contrast, the intra-die fluctuations consisting of both random and deterministic variations produce non-uniform electrical characteristics across the chip [16]. As one example of these fluctuations, we can think of the variations of photoresist thickness of inter-die and intra-die [16]. We can find that the fluctuation in the photoresist thickness seems random in lot-to lot, wafer-to-wafer and die-to-die [16]. However, the fluctuations within the die can be both deterministic and random. In such a way, the fluctuations of electrical parameters of inter-die and intra-die seem very complicated in the real semiconductor fabrication process. In this paper, using the concepts of the inter-die and intra-die fluctuations in the semiconductor manufacturing process [16], we try to perform the statistical analysis on the inter-array and intra-array variations in memristor crossbar array. Because the fabrication process of memristor crossbars is largely similar with the semiconductor process, we can possibly apply the statistical analysis method of the semiconductor process to the fluctuations of electrical parameters in memristor crossbars.

For the statistical simulation, in this paper, we considered four possible cases of inter-array and intra-array correlations. First, both the inter-array and intra-array correlations are assumed 0. It means that two arrays are not correlated at all and memristance values in each array are random. Second, the inter-array correlation is 0 and the intra-array 1. Here, two arrays are independent, while memristance values in each array are changed in the same way. Third, the inter-array correlation is 1 and the intra-array 0. Two arrays are correlated, but memristance values in each array are varied in random. Fourth, both the inter-array and intra-array correlations are 1. It is understood that the two arrays are correlated and memristance values in each array are varied in the same way.

### Tested Images

**Fig. 4:**
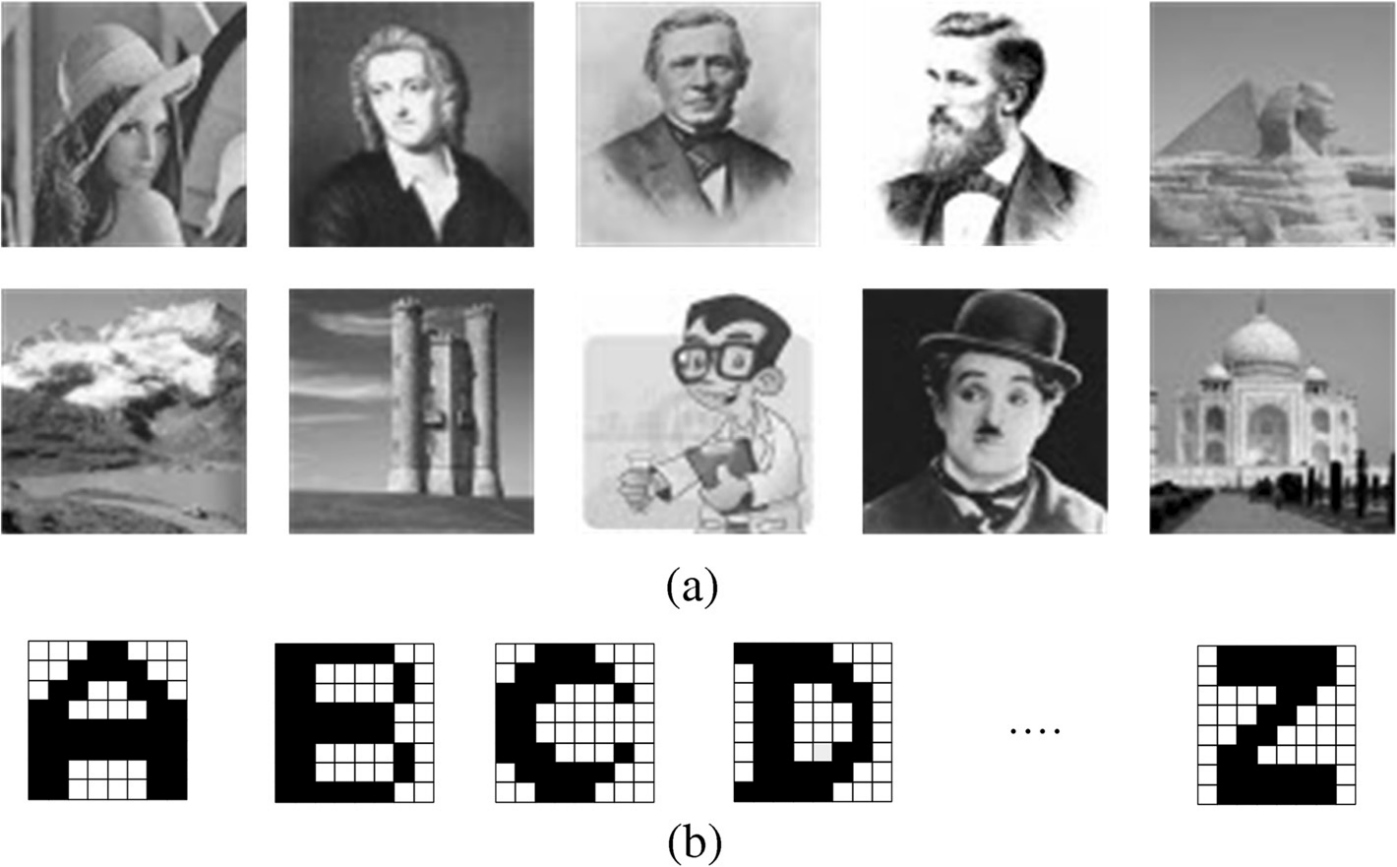
**a** 10 greyscale images with 32 × 32 pixels. **b** 26 black-and-white alphabet characters with 8 × 8 pixels

In the simulation, we tested 10 greyscale images with 32 × 32 pixels which are shown in Fig. 4a. We also tested 26 black-and-white alphabet characters with 8 × 8 pixels, as shown in Fig. 4b.

### Block Diagram of the New Twin Crossbar

**Fig. 5:**
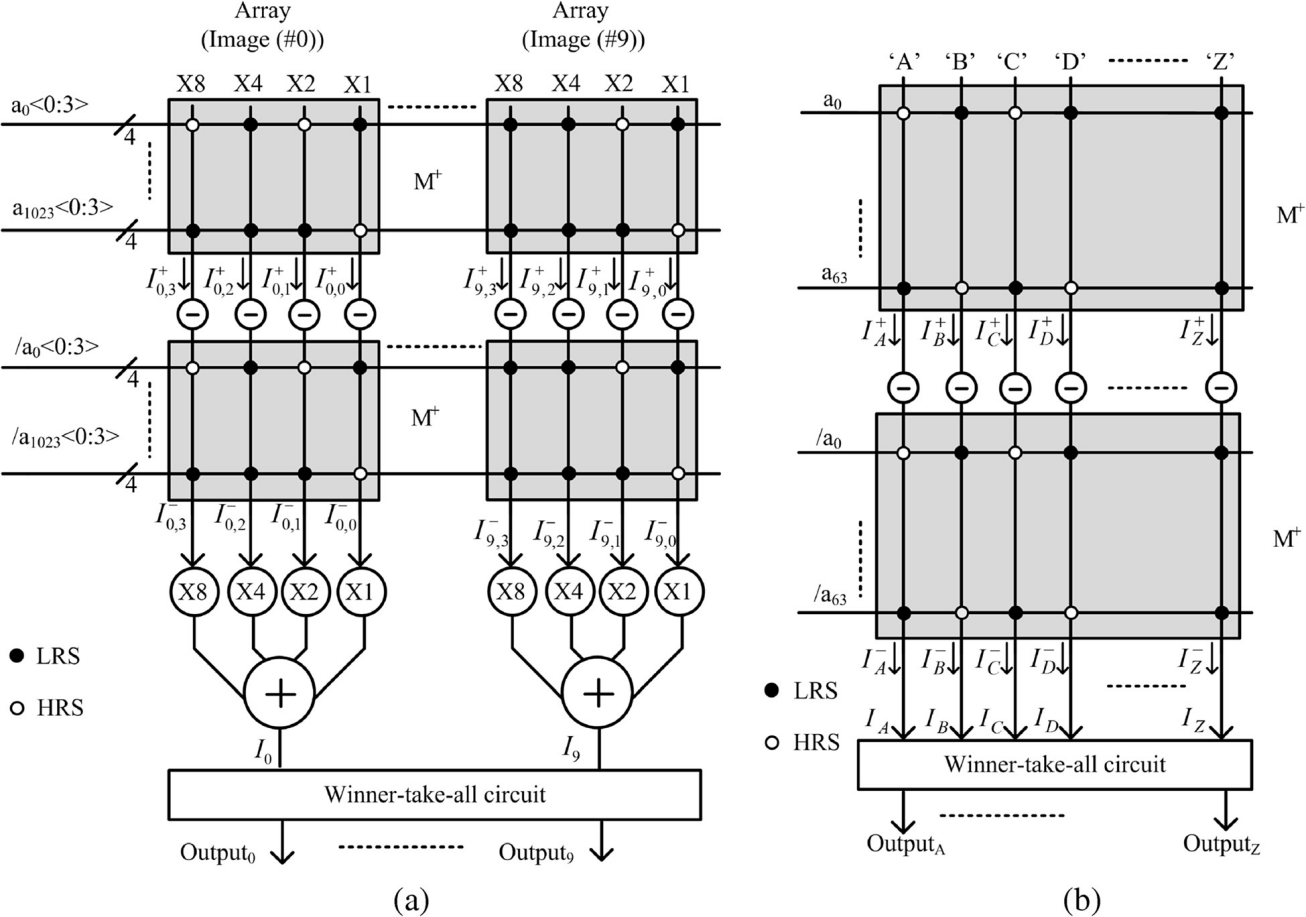
**a** Twin crossbar circuit of binary memristors for recognizing 10 greyscale images with 32 × 32 pixels [10]. **b** Twin crossbar circuit of binary memristors for recognizing 26 black-and-white alphabet characters with 8 × 8 pixels

Figure 5a shows a block diagram of the twin crossbar architecture of binary memristors for recognizing 10 greyscale images with 32 × 32 pixels [10]. The conceptual schematic of Fig. 5a was already shown in Fig. 2b. In Fig. 5a, as mentioned just earlier, the input is a greyscale image with 32 × 32 pixels [10]. Hence, the number of input pixels to the crossbar is 32 × 32 = 1024. Each pixel is digitized by 4 bits. Here, *a*_0_<0:3> is the 4-bit digitized inputs of *a*_0_. /*a*_0_<0:3> is the inversion of *a*_0_<0:3> [10]. In Fig. 5a, *a*_0_<0:3> is applied to the upper M^+^ array and /*a*_0_<0:3> is applied to the lower M^+^ array. The upper M^+^ array and the lower M^+^ array are identical to each other in Fig. 5a.

Among the 4-bit digitized signals of *a*_0_<0:3>, *a*_0_<3> is connected to the column with the weight as large as 8 in the upper M^+^ array. In Fig. 5a, ‘X8’ means the weight is as large as 8 and ‘X1’ means the weight is as small as 1. Similarly, /*a*_0_<3> is connected to the column of weight X8 in the lower M^+^ array. Using the simple current mirror circuit, we can perform the weighted summation with four weights of ‘X8’, X4’, ‘X2’ and ‘X1’. *I*_0_ is the result of weighted summation for the first image, #0, in Fig. 5a. *I*_0_ can be calculated as 8(*I*_0,3_^+^ − *I*_0,3_^−^) + 4(*I*_0,2_^+^ − *I*_0,2_^−^) + 2(*I*_0,1_^+^ − *I*_0,1_^−^) + (*I*_0,0_^+^ − *I*_0,0_^−^) for the input image (#0) with 32 × 32 pixels. *a*_0_ to *a*_1023_ and /*a*_0_ to /*a*_1023_ represent the input image and its inversion with 1024 (32 × 32) pixels. If the input image is similar with the stored image (#0), *I*_0_ becomes large. If they are very different each other, *I*_0_ becomes small. In Fig. 5a, we assume that the crossbar stores 10 images from #0 to #9. The amount of *I*_1_ indicates the similarity between the input image and the stored image (#1). Similarly, *I*_9_ means the similarity between the stored image (#9) and the input image. The winner-take-all circuit can choose one stored image that is the best match with the input image by comparing the 10 currents from *I*_0_ to *I*_9_ in Fig. 5a [10].

Similarly with Fig. 5a, Fig. 5b shows the block diagram of the twin crossbar circuit of binary memristors for recognizing 26 black-and-white alphabet characters with 8 × 8 pixels.

### Training Process of the Crossbar

**Fig. 6:**
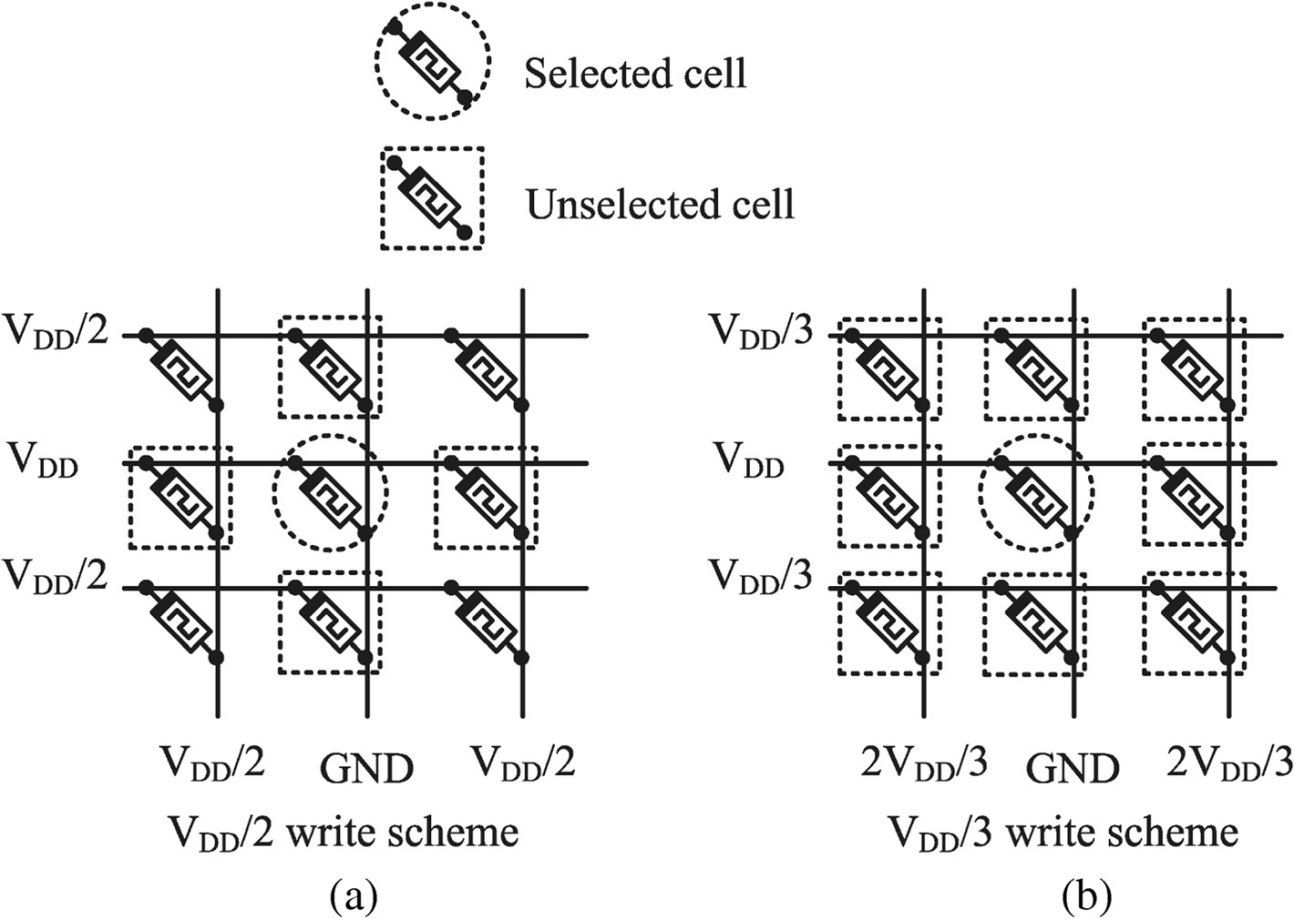
Memristor crossbar array write schemes. **a** 1/2*V*_DD_ write scheme. **b** 1/3*V*_DD_ write scheme [17]

The training of the crossbar circuit means changing memristor resistance value to HRS or LRS. Here, we can use the 1/2*V*_DD_ write scheme or 1/3*V*_DD_ write scheme [17] in training memristors to have the target resistance values of HRS and LRS in this paper. Figure 6a shows the 1/2*V*_DD_ write scheme, where the selected cell is applied by *V*_DD_ and GND. Here, the unselected cells on the same row or column with the selected cell are driven by 1/2*V*_DD_. If the resistance change due to this 1/2*V*_DD_ is much smaller than the resistance change due to the full *V*_DD_, the unselected cells with 1/2*V*_DD_ can keep their resistance values unchanged during the training process. If the unselected cells which should be driven by 1/2*V*_DD_ are very susceptible to this small voltage of 1/2*V*_DD_, we can use the 1/3*V*_DD_ write scheme, as shown in Fig. 6b. In this figure, the selected cell is applied by *V*_DD_ and GND, like the selected cell in Fig. 6a. However, the unselected cells in Fig. 6b are driven by only 1/3*V*_DD_ that is much smaller than the 1/2*V*_DD_ in Fig. 6a [17]. By doing so, we can suppress the unwanted resistance change of the unselected cells in Fig. 6b better than those in Fig. 6a.

## Results and Discussion

**Fig. 7:**
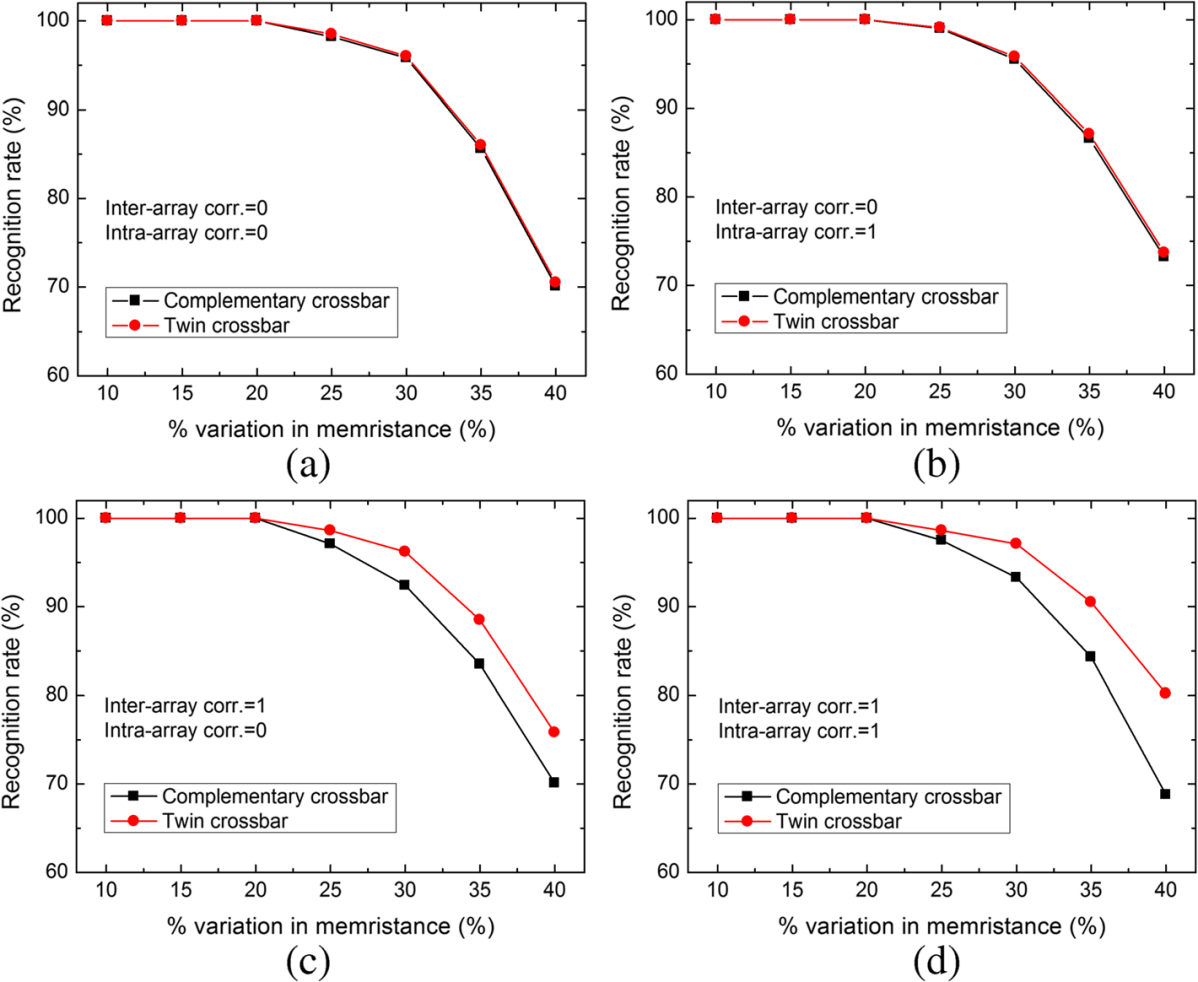
The comparison of recognition rate between the complementary and twin architectures for 10 greyscale images. **a** Inter-array correlation = 0 and intra-array correlation = 0. **b** Inter-array correlation = 0 and intra-array correlation = 1. **c** Inter-array correlation = 1 and intra-array correlation = 0. **d** Inter-array correlation = 1 and intra-array correlation = 1

In Fig. 7, we simulated the recognition rate for the 10 greyscale images with 32 × 32 pixels. In Fig. 7a, both the inter-array and intra-array correlations are assumed 0. In this case, the two crossbar architectures show the same rate in recognizing the tested images, because two arrays are not correlated with each other and all the memristors in each array have random variation. In Fig. 7b, the inter-array correlation is 0, but the intra-array correlation is 1. It means that two arrays are not correlated with each other, but all the memristors in each array have the correlation as strong as 1. Figure 7b also shows the same recognition rate for both the complementary array and the twin one, as already shown in Fig. 7a. From Fig. 7a, b, we can think that the complementary and twin architectures show the same recognition rate if two arrays are not correlated.

Now, let us see the case of inter-correlation = 1. Figure 7c shows the case of the inter-array correlation = 1 and the intra-array correlation = 0. In this case, the twin crossbar shows better recognition rate than the complementary crossbar. Here, the variation in two identical M^+^ arrays in the twin crossbar can be compensated, because two M^+^ arrays are correlated with each other. However, in the complementary crossbar, the variation in M^+^ array cannot be compensated by the variation in M^−^ array, because M^−^ array is the inversion of M^+^ array. By the same reason, we can also think that the twin crossbar can recognize the tested images better than the complementary crossbar in Fig. 7d. Both the inter-array and intra-array correlations are 1 in Fig. 7d. On average, the twin crossbar shows better recognition rate by 4 % than the complementary one for the inter-array correlation = 1 and intra-array correlation = 0. With the same amounts of variation in memristance, the twin architecture can recognize better by 5.6 % than the complementary one when both the inter-array and intra-array correlations are 1.

**Fig. 8:**
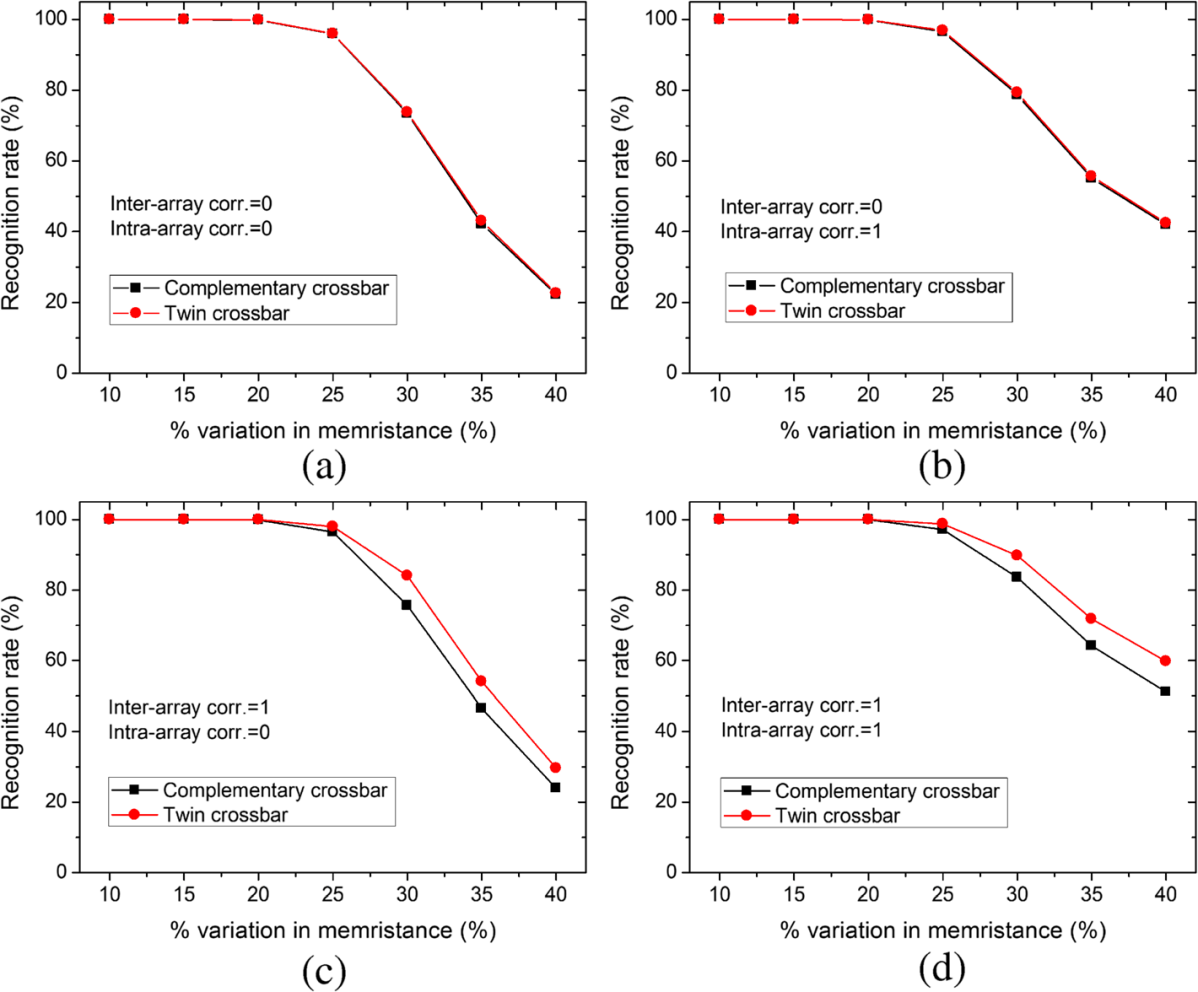
The comparison of recognition rate between the complementary and twin architectures for 26 black-and-white alphabet characters. **a** Inter-array correlation = 0 and intra-array correlation = 0. **b** Inter-array correlation = 0 and intra-array correlation = 1. **c** Inter-array correlation = 1 and intra-array correlation = 0. **d** Inter-array correlation = 1 and intra-array correlation = 1

In Fig. 8, we simulated the recognition rate for the 26 alphabet characters with 8 × 8 pixels. In more detail, in Fig. 8a the inter-array correlation = 0 and the intra-array correlation = 0. In Fig. 8b, the inter-array correlation = 0 and the intra-array correlation = 1. In Fig. 8c, the inter-array correlation = 1 and the intra-array correlation = 0. In Fig. 8d, the inter-array correlation = 1 and the intra-array correlation = 1. As shown in Fig. 8c, on average, the twin crossbar shows better recognition rate by 4.5 %. With the same amounts of variation in memristance, the twin architecture can recognize better by 6 % than the complementary one when both the inter-array and intra-array correlations are 1, as shown in Fig. 8d.

## Conclusions

This paper performed the comparative study on the statistical-variation tolerance between the two crossbars. Here, we compared the complementary crossbar architecture with the twin architecture for different variation and correlation parameters using the Monte Carlo method in Cadence Spectre software. To perform this comparative study, we tested 10 greyscale images and 26 black-and-white alphabet characters in terms of recognition rate with varying statistical-variation and correlation parameters.

As with the simulation results of 10 greyscale image recognitions, the twin crossbar shows better recognition rate by 4 % on average than the complementary one when the inter-array correlation = 1 and intra-array correlation = 0. When the inter-array correlation = 1 and intra-array correlation = 1, the twin architecture can recognize better by 5.6 % on average than the complementary one.

Similarly, when the inter-array correlation = 1 and intra-array correlation = 0, the twin architecture can recognize 26 alphabet characters better by 4.5 % on average than the complementary one. When the inter-array correlation = 1 and intra-array correlation = 1, the twin architecture is better by 6 % on average than the complementary one. By summary, we can conclude that the twin crossbar is more robust than the complementary one under the same amounts of statistical variation and correlation.
